# Increasing flooding frequency alters soil microbial communities and functions under laboratory conditions

**DOI:** 10.1002/mbo3.548

**Published:** 2017-11-07

**Authors:** Richard J. Randle‐Boggis, Peter D. Ashton, Thorunn Helgason

**Affiliations:** ^1^ Department of Biology University of York York UK

**Keywords:** climate change, DNA sequencing, flooding, metagenomics, microbial ecology

## Abstract

The impacts of increased flooding frequency on soil microbial communities and potential functions, in line with predicted environmental changes, were investigated in a laboratory‐controlled environment. More frequent flooding events altered microbial community composition and significantly increased the resolved species alpha‐diversity (Shannon index). The Bacteria:Archaea ratio was greater at the end of the experiment than at the start, more‐so after only one flood. Significant changes in taxa and functional gene abundances were identified and quantified. These include genes related to the reduction and oxidation of substances associated with anoxia, for example, those involved in nitrogen and sulfur cycling. No significant changes were observed in the methanogenesis pathway, another function associated with anoxia and which contributes to the emission of greenhouse gases.

## 1 INTRODUCTION

### 1.1 Climate change and flooding

It is predicted that climatic changes will increase the frequency of extreme precipitation events in places such as Northern Europe, North America, and Asia, particularly in winter, and that this will result in an increase in flooding frequency (Collins et al., 2013; Kirtman et al., 2013; Min, Zhang, Zwiers, & Hegerl, 2011). This will alter soil microbial ecosystems and biogeochemical cycles (e.g., N, C, Fe, and S), at least transiently. Complex microbial communities, such as those found in soil, can be highly responsive to environmental changes (Rinnan, Michelsen, Bååth, & Jonasson, 2007; Schmidt et al., 2000; Waldrop & Firestone, 2006).

### 1.2 Flooding and microbial ecosystems

Anoxic conditions resulting from flooding will affect soil properties and ecosystems (Ponnamperuma, 1984; Stams & Plugge, 2010). Zhou et al. (2002) reported that soils saturated in water have reduced bacterial diversities. Microorganisms dominate most biogeochemical cycles, and alterations to community structure and function may result in changes to these cycles. As the frequencies of extreme weather conditions are predicted to increase, it is necessary to understand how these changes will affect ecosystems and their functions.

Some studies research the effects of flooding on microbial ecosystems using targeted approaches. Studying four sites with varying flooding patterns along a river, Bodelier, Bar‐Gilissen, Meima‐Franke, and Hordijk (2012) discovered that the abundance of methanotrophs increased with the increase in flooding using denaturing gel gradient electrophoresis (DGGE) and phospholipid fatty acid analysis (PLFA). Kemnitz, Chin, Bodelier, and Conrad (2004) identified an increase in methanogen diversity in samples from the same river using terminal‐restriction fragment length polymorphism (T‐RFLP). Unger, Kennedy, and Muzika (2009) found that flooding decreased the bacteria:fungi ratio using PLFA. These studies provide a useful insight into the effects of flooding on microbial diversity and community composition; however, it is clear that a deeper understanding of the impacts of environmental stressors on the whole community is required. Furthermore, a gene‐oriented analysis is required to understand the functional responses to flooding in a pasture field.

Alternating flooding and draining will perturb microbial communities, as the anoxia will kill or suppress some populations and allow others to develop (Denef et al., 2001). Cycling between the two states will inhibit the community from stabilizing with a predominantly aerobic or anaerobic population, and those that thrive will be able to tolerate both conditions. Flood duration will impact the community as redox potentials take time to decrease during anoxia (Mohanty et al., 2013; Wang, DeLaune, Patrick, & Masscheleyn, 1993), with denitrification occurring, then iron and sulfur reduction, then finally methanogenesis (Patrick & Jugsujinda, 1992; Reddy & Patrick, 1975). Drainage oxidizes these compounds again, increasing the redox potential and inhibiting downstream reduction processes. Baldwin and Mitchell (2000) found that nitrification and denitrification decreased after periods of desiccation but increased again after rewetting, and Morillas et al. (2015) found that increased dry/wetting frequency decreased nitrification.

Anaerobic soils may contain methanogens, Archaea that produce CH_4_ under strictly anaerobic conditions, and flooding could increase their populations (Conrad, 2007). Methanotrophs, found both aerobically and anaerobically, metabolize CH_4_. Methane has a 100‐year global warming potential that is 32 times greater than CO_2_ (Myhre et al., 2013), thus studying the factors that increase CH_4_ flux is essential for understanding climate change risks. Studies of rice paddies (Ratering & Conrad, 1998; Sigren, Lewis, Fisher, & Sass, 1997; Yagi, Tsuruta, Kanda, & Minami, 1996) found that short‐term drainage of floods resulted in a sharp decrease in CH_4_ emissions. This is expected because methanogens are intolerant to even low levels of oxygen (Conrad, 2007). However, once flooding re‐occurred, CH_4_ emissions were still suppressed. This may have been caused by the oxidation of reduced sulfate and ferric iron during drainage (Patrick & Jugsujinda, 1992) providing a fresh source of substrates for sulfate/iron reducing bacteria. These would outcompete methanogens for H_2_ and acetate (Conrad, 2007). How microbial communities will respond to frequent flooding and drainage on pasture soil is yet to be investigated.

While flooding induces anoxia in the bulk soil, the oxic state present during and after drainage may restore the community to its previous state. Ponnamperuma (1984) reported that most of the changes to the physical, chemical, and biological processes of soil in response the flooding are reversed with draining and drying, however, the rate at which this occurs depends on many factors, such as the proliferation rates of species, redox potentials, the quantities of metabolic substrates present, and the flood subsidence rate. Obligate aerobic and facultative anaerobic bacteria grow best in aerobic conditions, but some can survive periods of hypoxia or anoxia, for example, *Methylosinus trichosporium* (Roslev & King, 1994) and *Mycobacterium smegmatis* (Berney, Greening, Conrad, Jacobs, & Cook, 2014). Frequent flooding interspersed with drainage periods will therefore only inhibit the growth of many bacteria species, rather than kill them. Furthermore, as a moist environment is preferable for many aerobic bacterial species (Fredrickson et al., 2008; Potts, 1994; Roberson, Chenu, & Firestone, 1993), occasional flooding will provide a suitable environment for these species during drained periods.

### 1.3 Aims and hypotheses

We investigate the impacts of increased laboratory‐controlled flooding frequency on microbial communities and their functions. We hypothesize that increased flooding frequency will significantly change the composition and decrease alpha‐diversity of microbial communities and their potential functions. Significant increases in abundances of genes involved in methane production and sulfate reduction are predicted following greater flooding frequencies, with decreases in methane oxidation.

## 2 EXPERIMENTAL PROCEDURES

### 2.1 Experimental design

Topsoil was collected from within a pasture field in Wiltshire located next to a river confluence (Lat. 51.044770, Long. −2.111945). The soil was collected away from the river and hedges. The soil association is Wickham 2: fine loamy over clayey soil (Figure Supporting Information) (National Soil Resources Institute (NSRI), 2013). The mean air temperature for the area is 10.0°C and the mean rainfall is 770.4 mm (Met Office, 2017).

The soil was passed through a 6 mm sieve and stored at room temperature for 1 week. It was then homogenized and placed in six 8 (hr) × 10 (d) cm plastic pots (700 g per pot).

### 2.2 Treatment

All replicates were subjected to an initial flood for 2 weeks. The pots were placed in open 1.8 L containers and filled with deionized water to a soil‐surface depth of 20 mm. The experiment was conducted in complete darkness at 18°C. After 2 weeks, all replicates were drained and their GWC brought to field capacity. For the remainder of the experiment, the 1 × flood treatments were not flooded again. The 3× flood treatments were left drained for 2 weeks, then subjected to two further 2‐week flooding treatments, with a 2‐week period in between and at the end where they were left to drain freely (Table 1).

**Table 1:** The treatment regime for the laboratory experiment

Time period (weeks)	1 × flood	3 × floods
12	*Saturation*	*Saturation*
34	Drained	Drained
56	Drained	*Saturation*
78	Drained	Drained
910	Drained	*Saturation*
1112	Drained	Drained

### 2.3 DNA sampling

Three randomly selected soil samples (2 g) were extracted from the homogenized soil prior to filling the treatment containers, representing the starting soil community. At the end of the experiment, soil samples were randomly extracted from each container from a depth of 5 cm using a 2 cm corer. DNA was extracted within 2 hr of sample collection using a PowerSoil^®^ DNA Isolation kit (250 mg) (Mo Bio Laboratories Inc., Carlsbad, CA, USA) following the manufacturer's protocol and stored at −80°C.

### 2.4 Sequencing

DNA quantities were determined using a Qubit^®^ Fluorometer (Life Technologies Corporation, Carlsbad, CA, USA) and the technical extraction replicates were pooled together in equal quantities to form biological replicates. Samples were purified using an Agencourt AMPure XP bead clean‐up kit following the manufacture's protocol (Beckman Coulter (UK) Ltd., High Wycombe, UK). Samples with concentrations greater than 10 ng/μl were diluted 1:10 using RNase‐free water to ensure that the quantities were appropriate for use with the Nextera XT DNA sample preparation kit (Illumina UK, Little Chesterfield, UK); concentrations too high result in fragment lengths that are too long for sequencing. The samples were further diluted with RNase‐free water to make 5 μl of solution with approximately 10 ng of DNA. DNA libraries were produced using the Nextera XT DNA sample preparation kit following the manufacture's protocol. The samples were pooled, resulting in a DNA concentration of 17.5 ng/μl. The libraries were sequenced using a MiSeq Personal Sequencer (Illumina UK, Little Chesterfield, UK) following the manufacturer's protocol. The v3 reagent kit was used, generating paired‐end reads of 600 bp.

### 2.5 Analyses

The paired‐end reads were merged with PEAR (Zhang, Kobert, Flouri, & Stamatakis, 2014). Unmerged forward reads were trimmed with Sickle (https://github.com/najoshi/sickle) using a mean phred score threshold of 25. Unmerged reverse reads were discarded to remove abundance bias when included with merged reads. The merged and trimmed forward read files were concatenated and uploaded to MG‐RAST (Table 2) (Meyer et al., 2008).

**Table 2:** Sample MG‐RAST IDs

Sample	Replicate	MG‐RAST ID
Start	1	4552009.3
Start	2	4552010.3
Start	3	4552011.3
1 × Flood	1	4552006.3
1 x Flood	2	4552007.3
1 x Flood	3	4552008.3
3 × Floods	1	4552012.3
3 × Floods	2	4552013.3
3 × Floods	3	4553069.3

Sequences were annotated with a representative hit annotation technique, which selects a single, unambiguous annotation for each feature. The RefSeq database was used for taxonomic identification and Subsystems for functional assignment. The maximum E‐value was 1 e^−15^, providing a strict search parameter. The minimum sequence identity was 60%, and the minimum alignment length was 20 bases. These parameters were selected to maximize annotation sensitivity without significantly sacrificing precision (Randle‐Boggis, Helgason, Sapp, & Ashton, 2016). Taxa and functions with a total abundance below five sequences across all samples were removed, as confident conclusions cannot be drawn for such low representations as there is a greater chance that they are influenced by annotation errors (Randle‐Boggis et al., 2016). Relative abundance values were generated and arc‐sin square root transformed to normalize the proportional data. Rarefaction curves (1,000 resamples) display the taxa richness per sequence count, visualizing the effectiveness of sequence coverage. The lowest taxonomic level studied was the order level. Below this we consider the proportion of potential incorrect annotations unacceptable (Randle‐Boggis et al., 2016).

The Bacteria:Archaea abundance ratio was calculated and the α‐diversity of each sample calculated using the Shannon index, an abundance‐weighted average of the logarithm of the relative abundances of taxa. Treatment dissimilarities were tested with Analysis Of Similarity (ANOSIM, 100,000 permutations), Principal Coordinates Analysis (PCoA) and hierarchical clustering, all using the Bray–Curtis dissimilarity method. Taxa and function PCoA weightings were ranked and plotted (Figure S4); those before or after the curve plateaus, at >0.02 or <−0.02, respectively, were considered for further analysis. Changes in relative abundance of orders and gene functions were analyzed using ANOVA. Multiple comparison corrections were made using Benjamini–Hochberg. Significant differences in the abundances of methanogenesis, CH_4_ oxidation and sulfur reduction genes were selectively tested for using ANOVA.

Data processing was performed in python (Python Software Foundation, v. 2.7.10) and statistical analyses in R (R Core Team, 2012, v. 3.0.3).

## 3 RESULTS

### 3.1 Sequencing

8,408,535 paired‐end sequences were generated with a mean sample sequence count of 934,300 ± 664,308. PEAR merged 78.98 ± 4.45% of reads. All samples maintained a mean phred score greater than 30 (Figure S1). The mean sequence length after merging and trimming was 231 ± 131 bases (Figure S2). The rarefaction curves suggest that sequence coverage was sufficient in all samples to represent the microbial community at the genus level; an enhanced sampling effort would yield only a few additional genera (Figure S3). Three x Floods replicate 1 would benefit the most from enhanced sampling.

### 3.2 Diversity and bacteria: Archaea ratio

There was a significant difference between the order α‐diversities of the samples (Start: 4.478 ± 0.010, 1 × Flood: 4.465 ± 0.005, 3 × Floods: 4.492 ± 0.007; ANOVA, *F* = 8.486, df = 2, *p* = .018). Post hoc testing revealed that the 3 × Floods treatment was significantly more diverse from than the 1× Flood treatment (Tukey's HSD, *p* = .015).

The Bacteria:Archaea ratio significantly increased in response to flooding (square‐root transformed (n:1): Start: 12.01 ± 0.15, 1 × Flood: 13.26 ± 0.31, 3 × Floods: 12.74 ± 0.11; ANOVA, *F* = 26.85, df = 2, *p* = .001; Tukey's HSD, Start & 1 × Flood: *p* = .001, Start & 3 × Floods, *p* = .012).

### 3.3 Sample dissimilarities

**Figure 1:**
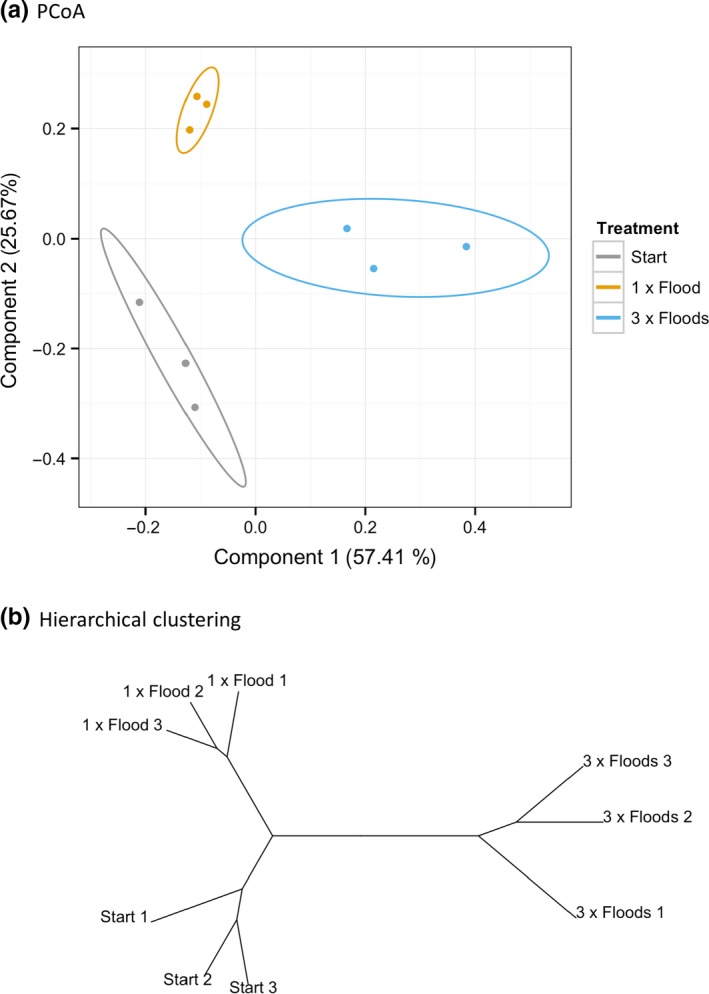
A PCoA (a) and hierarchical clustering analysis (b) of the relative abundance of orders (Bray–Curtis distance method). Ellipses in a display 95% confidence intervals

**Figure 2:**
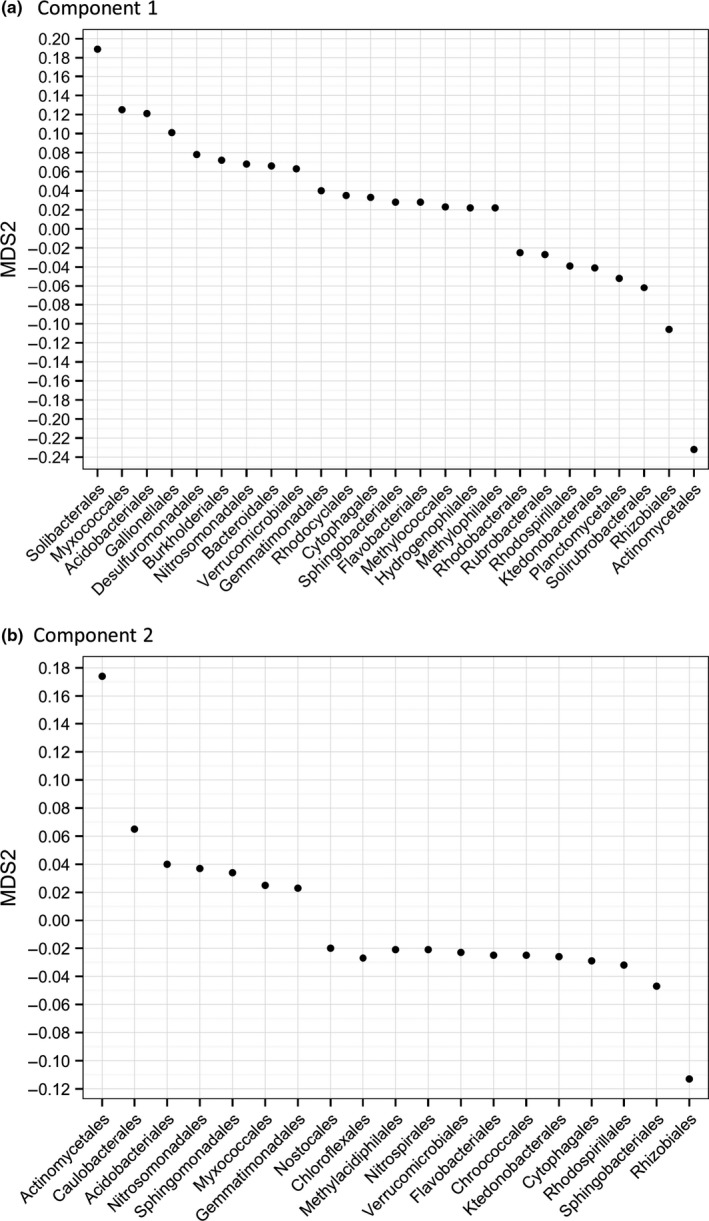
Orders with PCoA weightings > 0.02 or < −0.02 for component 1 (a) and component 2 (b) in the taxonomic PCoA

**Figure 3:**
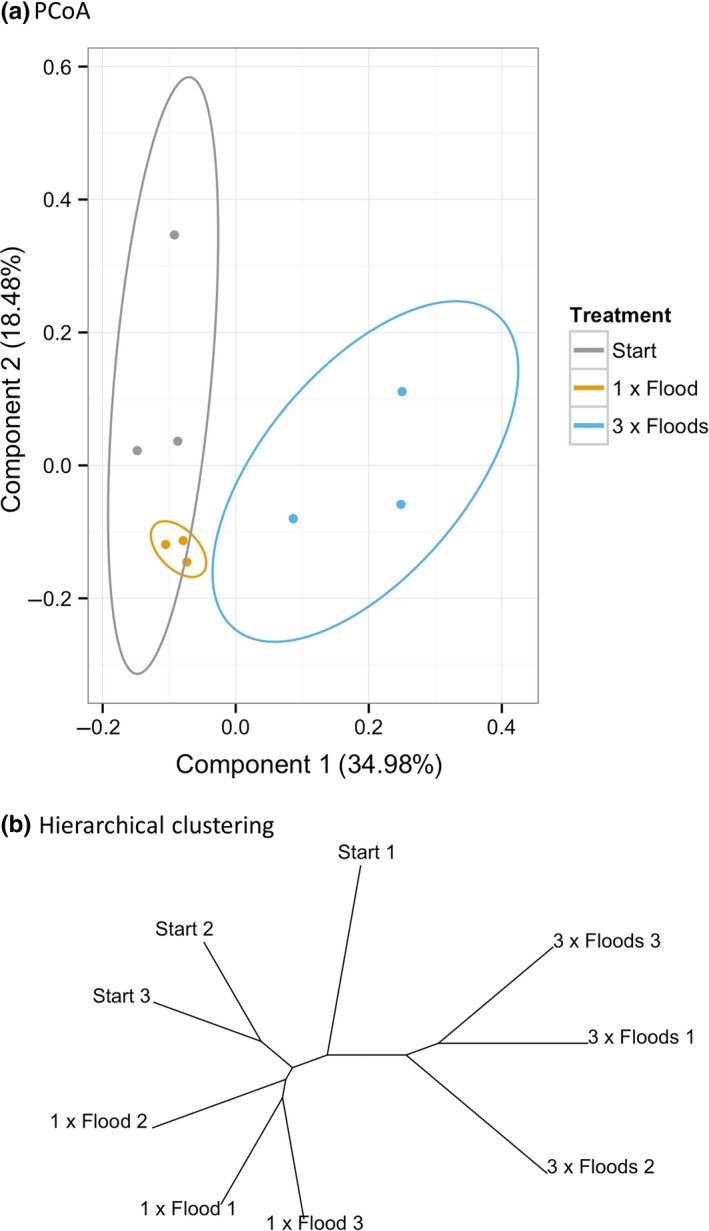
(a): A PCoA of potential level 3 functions (Bray–Curtis distance method). Ellipses display 95% confidence intervals. (b): A hierarchical clustering analysis of potential level 2 functions (Bray–Curtis distance method)

Flood frequency had a significant effect on the microbial community taxonomic composition (ANOSIM, R: 0.679, *p *=* *.023) and function (ANOSIM, R: 0.251, *p *=* *.003). Both treatments were taxonomically dissimilar from each other and the Start samples (Figures 1, 2). The 3 × Floods samples were functionally dissimilar from the Start and from 1 × Flood samples, both of which were not dissimilar from each other (Figure 3, Tables 3 and 4).

**Table 3:** Functions with PCoA weightings >0.02 or <−0.02 for component 1 in the functional PCoA

Function	MDS1
Cobalt‐zinc‐cadmium resistance	0.042
Ton and Tol transport systems	0.040
Flagellar motility	0.036
Bacterial Chemotaxis	0.035
Hydrogenases	0.030
Iron acquisition in vibrio	0.029
Sugar utilization in thermotogales	0.026
Lactose and galactose uptake and utilization	0.026
C jejuni colonization of chick caeca	0.025
Lactose utilization	0.024
Zinc resistance	0.022
Two‐component regulatory systems in campylobacter	0.021
Nitrosative stress	0.021
Outer membrane	0.021
Respiratory complex I	0.021
Major outer membrane proteins	0.020
General secretion pathway	0.020
Siderophore pyoverdine	0.020
Phospholipid and fatty acid biosynthesis‐related cluster	0.020
Niacin‐choline transport and metabolism	−0.020
Coenzyme PQQ synthesis	−0.020
Cobalamin synthesis	−0.021
Proline, 4‐hydroxyproline uptake and utilization	−0.021
Amidase clustered with urea and nitrile hydratase functions	−0.021
Glutathione analogs: mycothiol	−0.021
Iojap	−0.024
Creatine and creatinine degradation	−0.027
Phage integration and excision	−0.028
cAMP signaling in bacteria	−0.033
CBSS‐222523.1.peg.1311	−0.033
CO Dehydrogenase	−0.043
CBSS‐314269.3.peg.1840 (CO Dehydrogenase proteins)	−0.044

**Table 4:** Functions with PCoA weightings >0.02 or <−0.02 for component 2 in the functional PCoA

Function	MDS2
CBSS‐222523.1.peg.1311	0.034
cAMP_signaling_in_bacteria	0.034
Iojap	0.034
Cluster_with_phosphopentomutase_paralog	−0.020
SigmaB_stress_responce_regulation	−0.024

### 3.4 Taxonomic and functional abundances

**Figure 4:**
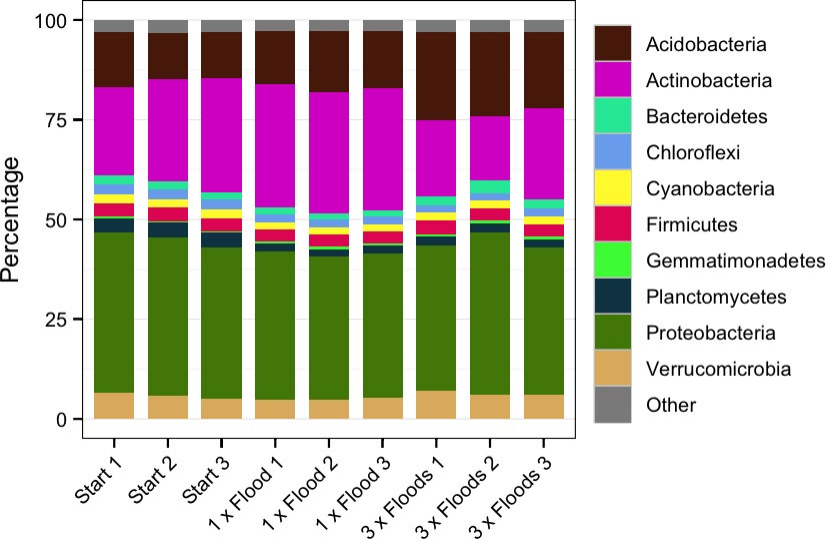
The relative abundances of phyla in each sample

The most abundant phyla across the samples were Proteobacteria, Actinobacteria, Acidobacteria, and Verrucomicrobia (Figure 4), which are often dominant phyla (Janssen, 2006).

The relative abundances of 29 orders (out of 223) were significantly different among the treatments, after correcting *p*‐values for multiple comparison corrections (ANOVA and Benjamini–Hochberg) (Table 5). Most significant differences occur between the 1 × Flood treatment and the 3 × Floods (Tukey's HSD). There were no significant differences between Subsystems level 3 functions, however, at level 2, 14 out of 166 functions were significantly different (Table 6).

**Table 5:** The orders with significantly different relative abundances between the samples (ANOVA)

Order	Start (x̄)	1 × F (x̄)	3 × F (x̄)	Corrected *p*‐value	Tukey's HSD		
					Start/1F	Start/3F	1 × F/3 × F
Rhodospirillales	0.014	0.013	0.012	<.001	<0.001	<0.001	<0.001
Planctomycetales	0.020	0.015	0.016	<.001	<0.001	<0.001	0.002
Myxococcales	0.016	0.018	0.022	.001	0.035	<0.001	<0.001
Sphingomonadales	0.008	0.009	0.008	.008	<0.001	*N.S*.	<0.001
Rhizobiales	0.046	0.041	0.039	.010	0.002	<0.001	*N.S*.
Enterobacteriales	0.007	0.007	0.007	.011	0.001	*N.S*.	0.002
Caulobacterales	0.008	0.011	0.009	.012	<0.001	0.014	0.015
Rhodobacterales	0.013	0.012	0.012	.012	0.006	<0.001	0.025
Solibacterales	0.026	0.028	0.034	.013	*N.S*.	0.001	0.003
Nitrospirales	0.006	0.005	0.005	.013	0.001	*N.S*.	0.003
Acidobacteriales	0.016	0.019	0.022	.014	0.033	<0.001	0.005
Nostocales	0.007	0.007	0.007	.015	0.001	*N.S*.	0.003
Fibrobacterales	0.001	0.001	0.002	.017	*N.S*.	0.001	0.004
Desulfobacterales	0.005	0.005	0.006	.017	*N.S*.	0.016	0.001
Chroococcales	0.011	0.010	0.010	.020	0.001	*N.S*.	0.012
Burkholderiales	0.023	0.024	0.026	.022	*N.S*.	0.002	0.009
Syntrophobacterales	0.006	0.006	0.007	.023	0.026	*N.S*.	0.002
Sphaerobacterales	0.006	0.006	0.005	.025	0.039	0.002	*N.S*.
Cytophagales	0.008	0.007	0.008	.031	0.039	*N.S*.	0.003
Desulfovibrionales	0.007	0.006	0.007	.034	0.037	*N.S*.	0.003
Chlorobiales	0.006	0.006	0.006	.039	0.032	*N.S*.	0.005
Chloroflexales	0.010	0.009	0.009	.040	0.007	0.010	*N.S*.
Oscillatoriales	0.006	0.005	0.006	.040	0.005	*N.S*.	0.021
Alteromonadales	0.007	0.006	0.007	.040	0.041	*N.S*.	0.005
Nitrosomonadales	0.006	0.008	0.009	.041	*N.S*.	0.005	*N.S*.
Lentisphaerales	0.002	0.002	0.002	.041	*N.S*.	0.018	0.007
Rubrobacterales	0.007	0.007	0.006	.041	*N.S*.	0.005	0.017
Gemmatimonadales	0.006	0.007	0.008	.043	*N.S*.	0.006	*N.S*.
Actiniaria	0.001	0.001	0.001	.049	*N.S*.	*N.S*.	0.007
Chromatiales	0.008	0.008	0.008	<.050	*N.S*.	0.049	0.008

The *p*‐values are adjusted for multiple comparisons (Benjamini–Hochberg) and post hoc tests were performed (Tukey's HSD). *N.S*., not significant.

**Table 6:** The level 2 Subsystems functions with significantly different relative abundances between the samples (ANOVA)

Function	Start (x̄)	1 × F (x̄)	3 × F (x̄)	Corrected *p*‐value	Tukey's HSD		
					Start/1F	Start/3F	1 × F/3 x F
Monosaccharides	0.012	0.012	0.013	.007	0.019	<0.001	<0.001
ABC transporters	0.008	0.008	0.008	.008	0.001	<0.001	*N.S*.
Resistance to antibiotics and toxic compounds	0.017	0.016	0.018	.010	0.043	0.002	<0.001
Peripheral pathways for catabolism of aromatic compounds	0.010	0.010	0.010	.010	*N.S*.	0.001	<0.001
Bacterial cytostatics differentiation factors and antibiotics	0.001	0.002	0.001	.011	0.008	0.012	<0.001
Phages Prophages	0.011	0.010	0.010	.020	0.025	0.001	0.019
Nucleotidyl phosphate metabolic cluster	0.009	0.008	0.008	.022	0.004	0.001	*N.S*.
Cytochrome biogenesis	0.005	0.005	0.005	.033	*N.S*.	0.006	0.002
Molybdopterin oxidoreductase	0.003	0.003	0.003	.035	*N.S*.	0.005	0.003
Capsular and extracellular polysacchrides	0.011	0.011	0.011	.036	*N.S*.	0.030	0.002
Toxins and superantigens	0.001	0.001	0.001	.037	*N.S*.	0.002	0.013
Organic sulfur assimilation	0.008	0.007	0.007	.038	0.021	0.003	*N.S*.
Tricarboxylate transporter	0.003	0.003	0.003	.040	0.002	*N.S*.	*N.S*.
Metabolism of central aromatic intermediates	0.008	0.008	0.008	.040	*N.S*.	0.005	0.007
Alpha proteobacterial cluster of hypotheticals	0.002	0.001	0.001	.049	*N.S*.	0.004	*N.S*.

The *p*‐values are adjusted for multiple comparisons (Benjamini–Hochberg) and post hoc tests were performed (Tukey's HSD). N.S., not significant.

**Figure 5:**
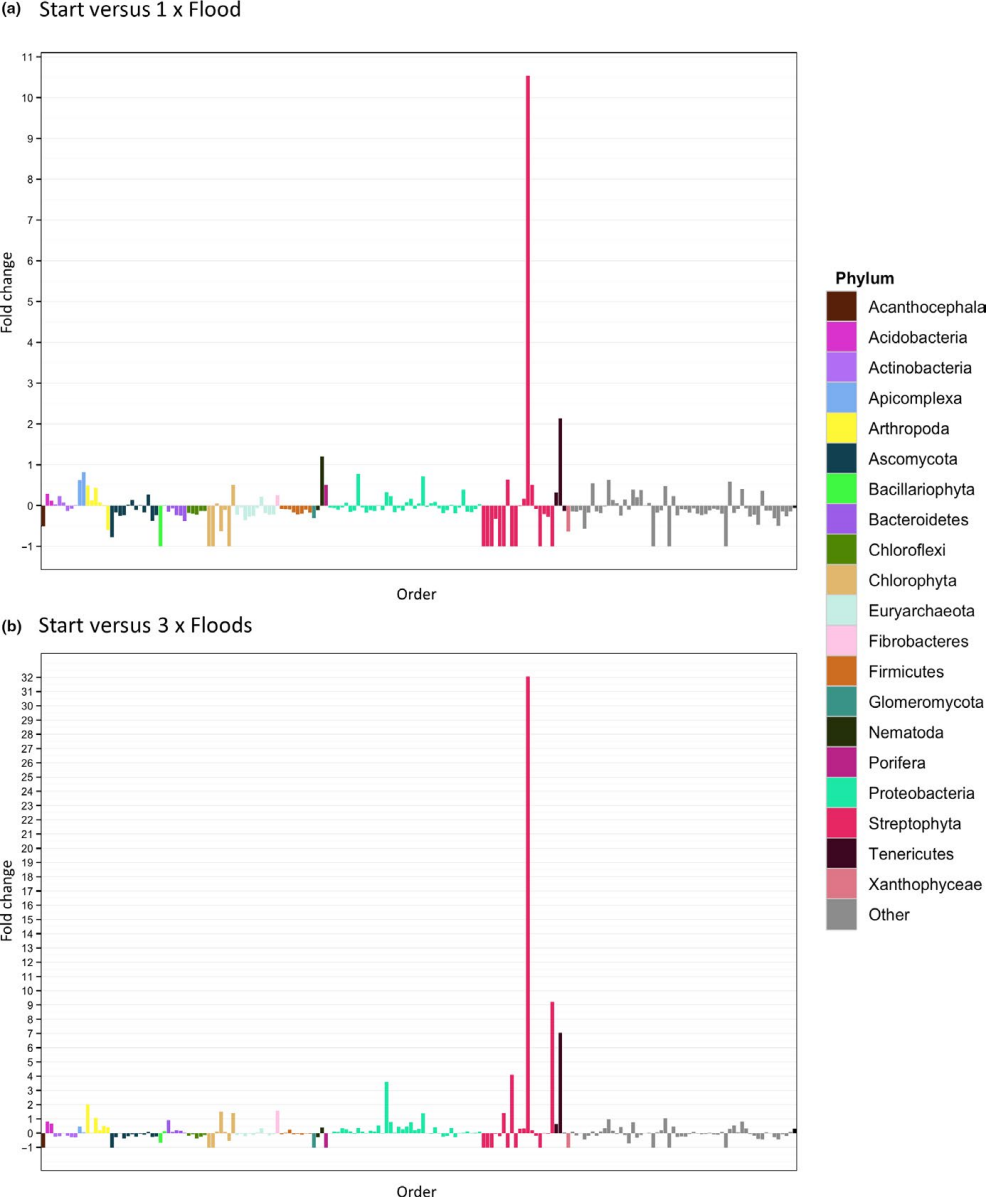
The fold changes of orders, colored by phyla, between the Start and 1 × Flood (a) and the Start and 3 × Floods (b) treatments

Of the 206 orders detected at the start, 66 population relative abundances increased, 122 population relative abundances decreased and 18 populations were undetected after receiving one flood (Table S1). 107 increased, 78 decreased and 21 were undetected after receiving three floods (Table S2). 17 orders were undetected in the starting soil but were detected at the end of the experiment. Tables S3 and S4 show the fold changes between orders (Figure 5). Of the 1,080 level 3 functions detected at the start, 537 relative abundances increased, 471 relative abundances decreased and 46 relative abundances were undetected after receiving one flood (Table S5). 512 level 3 functions increased, 483 decreased and 46 were undetected after receiving three floods (Table S6). 39 level 3 functions were undetected in the starting soil but were detected at the end of the experiment.

### 3.5 Relative abundance of selected functional groups

**Figure 6:**
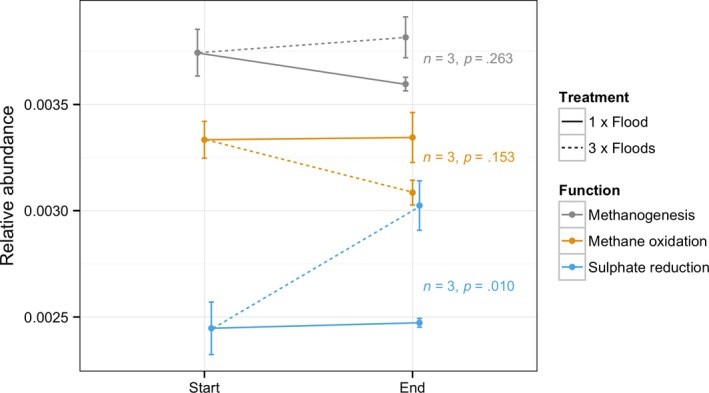
The differences in relative abundances of genes involved in methanogenesis, methane oxidation, and sulfur reduction (ANOVA). Error bars show standard deviation

The relative abundances of genes involved in methanogenesis and CH_4_ oxidation were not significantly different (ANOVA, methanogenesis: *F* = 1.681, df = 2, *p* = .263; CH_4_ oxidation: *F* = 2.535, df = 2, *p* = .159). There was a significant difference in the relative abundances of genes involved in sulfate reduction (ANOVA, *F* = 11.07, df = 2, *p* = .001, Tukey's HSD: Start & 3 × Floods: *p* = .014, 1 × Flood & 3 × Flood: *p* = .017) (Figure 6).

## 4 DISCUSSION

### 4.1 Diversity and bacteria: Archaea ratio

The order α‐diversities were significantly greater in the samples that received three floods than those that received one. While it is hypothesized that anaerobic environments would tend toward a lower α‐diversity over time, the short‐term repeated shift between aerobic and anaerobic conditions would inhibit a community from stabilizing, allowing populations of facultative anaerobic organisms to develop and aerobic populations to recover after flooding. Pett‐Ridge and Firestone (2005) and Frindte, Allgaier, Grossart, and Eckert (2015) discuss how redox fluctuations structure microbial communities in tropical soils, and Forth, Liljebladh, Stigebrandt, Hall, and Treusch (2015) report how anoxic microbial communities shift toward those found in oxic conditions upon oxygenation. Our results may partly be due to community shifts over time, and stochastic shifts are not accounted for. The differences between the treatments, however, indicate the effects of increased flooding frequency.

The Bacteria:Archaea ratio increased in response to flooding, with the greatest ratio observed in the 1 × Flood treatment. The relative abundance of archaea decreased. Archaeal RNA polymerase initiation factors and archaeal thermosomes, involved in transcription and protein structuring, respectively, also decreased. Most archaea are either strict anaerobes or can only tolerate low levels of oxygen (Berg et al., 2010), thus the drainage period in between the floods would kill several of the strict anaerobes. Some bacteria, on the other hand, can survive periods of hypoxia or anoxia (Berney et al., 2014; Roslev & King, 1994) and some would thrive in the moist environment provided by the initial flood (Fredrickson et al., 2008; Potts, 1994; Roberson et al., 1993).

### 4.2 Sample dissimilarities

The community compositions were all dissimilar, revealing that flood frequency has a strong impact on community structure; the 3 × Flooded communities were the most distinct. This was expected, as soil microbial communities can be highly responsive to environmental changes (Rinnan et al., 2007; Schmidt et al., 2000; Waldrop & Firestone, 2006). While the 3 × Flooded communities were functionally different from the other two, the start and 1 × Flood communities were not significantly different. The discrepancy between taxonomic and functional results is most likely due to functional complementarity among taxonomically different microbial communities, a reminder that taxonomic fluctuations do not necessarily imply functional shifts. The 3 × Flood samples are less aerobically stable than the other samples and experience greater perturbation, thus communities will undergo a much greater shift that would likely include significant functional differences.

### 4.3 Taxonomic and functional shifts

Orders that decreased in response to both treatments include several eukaryotes such as fungi (Capnodiales, Mucorales, and Polyporales) and algae (Cyanidiales). As this was a controlled laboratory experiment using homogenized soil, the loss of free organic matter due to consumption may cause the populations of many fungi and algae to decrease. Algae orders Chroococcales and Oscillatoriales both decreased after receiving one flood, but not three. The repeated flooding would limit the effects of desiccation between floods, allowing organisms that prefer moist environments to survive.

Most bacteria involved in the nitrogen cycle that declined in abundance decreased in response to one flood and not to three floods. These include Enterobacteriales, which are largely facultative anaerobes and nitrate reducers (Imhoff, 2005), and Nitrospirales and Nostocales, both aerobic nitrifying bacteria. Furthermore, the relative abundances of RNA polymerase sigma‐54 factor RpoN, a function involved in nitrogen assimilation and fixation (Gardner, Gessner, & Gardner, 2003; Powell et al., 1995), and its response regulator both increased in response to three floods but not one, as did nitrosative stress genes. Rhizobiales populations, which include four nitrogen‐fixing families, declined in response to both treatments, whereas populations of nitrifying Nitrosomonadales increased in response to both treatments. Initial wetting releases nitrogen that becomes available for nitrification, but after long periods of desiccation several bacteria die off (De Groot & Van Wijck, 1993). As the initial influx of nitrites is assimilated there will be less available for nitrifying bacteria to oxidize. The abundance of genomes containing heterocyst (nitrogen‐fixing cells) formation genes in cyanobacteria increased in response to one flood; heterocysts are formed during nitrogen stress, supporting most of our taxonomic findings (with the exception of Nitrosomonadales). Rewetting allows oxidized material to be reduced again, continuing the cycle, and the additional periods of anoxia will permit denitrification (Baldwin & Mitchell, 2000). Verhoeven, Laanbroek, Rains, and Whigham (2014) discovered a decrease in nitrification and denitrification in mangroves after increased flooding frequency, opposing these findings. Nutrient cycling is influenced by a variety of factors, for example: nutrient availability, redox potential, microbial community composition, and temperature. Discrepancies between results are therefore expected due to differing conditions, such as those in saline mangroves versus those in terrestrial pasture soils.

We predicted that methanogen and methanotroph populations would increase in response to a greater flooding frequency, but the patterns were complex. The methanotrophic family Methylococcaceae within the Rhizobiales, which are important oxidizers of CH_4_ in flooded soils (Conrad, 1996) declined in response to both treatments, as did all the other families in this order. Methanotrophic Methylococcales populations, however, did increase as predicted after three floods. Methanotrophic Rhizobiales can survive anaerobic conditions, so it is expected that their populations would also have increased in response to flooding and associated CH_4_ emissions. One explanation for the relatively weak response of methanotrophs to flooding may be that a corresponding increase in methanogen populations was not observed (and indeed the proportion of archaea also declined, against our original prediction). Therefore, an increase in CH_4_ production may not have occurred, resulting in little response from methanotroph populations such as Methylococcaceae. In fact, genes involved in the serine–glyoxylate cycle, a part of methylotrophic metabolism (Ensign, 2006), decreased in response to three floods. The lack of developing methanogen populations could be explained by the observed increase in sulfate‐reducing bacteria after three floods, as these initially out‐compete methanogens for substrates to metabolize (Conrad, 2007).

The greater taxonomic resolution achievable by NGS, compared to other methods such as DGGE and T‐RFLP, allows for more detailed understandings of ecosystems to be made. However, the complexity of interactions and responses means that environmental data such as nutrient content and gas fluxes is necessary to make reliable conclusions. To further understand the methanogen/methanotroph results discussed above, sulfur compound content, hydrogen content and CH_4_ fluxes need to be measured. This would verify the potential functional responses observed in the DNA.

Populations of several strict anaerobic organisms decreased after one flood followed by oxygenation, and many increased in response to three floods. Syntrophobacterales, Chlorobiales, Clostridiales, and Desulfovibrionales are all obligate anaerobes that decreased after the one flood treatment and increased after three floods. Chlorobiales oxidize sulfur compounds, H_2_ or Fe(II) (Bryant & Frigaard, 2006), and Desulfovibrionales reduce sulfates, thus are important in mineral cycling. Alkanesulfonate assimilation, involved in sulfur assimilation during limited sulfur availability (Ellis, 2011), decreased after three floods; this supports our taxonomic findings. Genes involved in organic sulfur assimilation decreased overall in response to both treatments.

The reduction in Fe(III) during the floods would likely have caused the increase in the Fe(II) oxidizing bacteria Gallionellales observed after both treatments, due to the spike in substrate availability (Conrad, 2007). Both treatments resulted in an increase in abundance of genes involved in iron acquisition, transport, and metabolism, with Ton and Tol transport systems (iron transport, (Noinaj, Guillier, Barnard, & Buchanan, 2010)) increasing after three floods only. The increase in reduced metals and other substrates due to repeated flooding would explain the increase observed in cobalt‐zinc‐cadmium resistance genes and substrate uptake regulation (e.g., Ton and Tol transport systems). These increases were not observed in the one‐flood samples, probably due to the resulting oxidation after drainage. Hydrogenase genes, largely involved in anaerobic metabolism (Vignais & Billoud, 2007), also increased after three floods. To further understand these interactions, the behavior of the microbes needs to be linked to detailed soil chemistry analysis.

Not all anaerobes decreased after the one‐flood treatment followed by desiccation; Rhodocyclales, which contains aerobic species but also anaerobic denitrifying oligotrophs (Brenner, Krieg, & Staley, 2007), increased after both treatments. Fibrobacteres, which include many anaerobic rumen bacteria (Ransom‐Jones, Jones, McCarthy, & McDonald, 2012), increased after three floods but did not change significantly after one flood. While the soil was homogenized for the experiment, localized microbial populations from feces may have been present.

Other orders that increased after both treatments include Euglyphida, Gemmatimonadetes, and Myxococcales. Euglyphida are amoebae common in soils, marshes, and organic‐rich environments that feed on bacteria (Lamentowicz et al., 2011). A meta‐analysis suggested that Gemmatimonadetes are adapted to arid conditions (DeBruyn, Nixon, Fawaz, Johnson, & Radosevich, 2011), suggesting this result is unexpected. However, Gemmatimonadetes typically make up 2.2% of soil bacteria (Janssen, 2006), and the only characterized species was isolated from wastewater (Zhang, 2003), thus presence in moist soils is surprising. The increase in Myxococcales hints at one of the current caveats of metagenomics. Myxococcales has an exceptionally long genome (ca. 13 mb) (Schneiker et al., 2007), so for a given number of individuals, sequence read abundance of large genome organisms is likely to be disproportionate and give a skewed impression of community structure. This could be accounted for using the genome sizes of all organisms present, but as yet, this information is not available for complex communities. This issue is exacerbated in eukaryotes, where not only are genomes typically much longer, but the frequency of genes and the functional complexity are not correlated with genome length—the so called C‐value paradox (Thomas, 1971).

Planctomycetes, Rhodobacterales, and Rhodospirillales decreased after both treatments. These are typically aquatic bacteria, and Rhodospirillales can use sulfide or hydrogen as an electron donor (sulfide is produced by sulfate reducing bacteria typically under anaerobic conditions (Barton, 1995), although they can function aerobically (Kjeldsen, Joulian, & Ingvorsen, 2004; Muyzer & Stams, 2008). We might expect that Planctomycetes, Rhodobacterales, and Rhodospirillales populations would increase in response to flooding due to the anoxic conditions and availability of reduced substrates. That this is not the case again suggests that a better understanding of the biology of these microbes is required and the chemical properties of the soil need to be studied throughout these experiments.

Many of the greater fold changes in relative abundances were attributed to mammals and insects, for example: Carnivora, Lagomorpha, Coleoptera, Hemiptera, and Phthiraptera. In the case of the mammals, this is likely to be from residual DNA in the soil, such as from rabbit skin. Increases in total abundance of residual DNA in a laboratory experiment are not surprising as, despite homogenization, spatial variation in relatively larger eukaryotic material is unavoidable; therefore it may not be accurately represented in the results. The soil was sieved and homogenized prior to the experiment, and no insects were observed. While some invertebrates are microscopic, caution should be taken before conclusions are made about these orders as the DNA observed could, like the mammals, be due to residual DNA rather than actual reflections of populations responding to the treatments. Without this critique of results, false conclusions about species’ responses to treatments could be made.

Genes involved in cell growth (RNA polymerase sigma‐70 factor), cell signaling (bacterial cAMP signaling), and membrane transport (ABC transporters) decreased after both treatments. The reduction in carbon input due to the removal of plants would restrict growth. Cell signaling is most beneficial when bacterial cell densities are at their highest (Darch, West, Winzer, & Diggle, 2012), so a reduction in that is expected too, as the carbon reduction and water stresses perturb populations. The reduction in membrane transport genes can due to the sieving, homogenization, and removal of plants reducing the amount of extracellular compounds being available for cell uptake, thus favoring species adapted to relatively lower nutrient environments (than in situ pasture soils).

Genes involved in flagellum motility and bacterial chemotaxis increased in response to three floods, but not one flood, suggesting a possible link between flooding frequency and bacterial mobility. Flooding changes the chemical composition of soil, prompting chemotaxis (Bren & Eisenbach, 2000). Transcriptomics would be advantages here to determine which genes are being expressed, rather than just observing which are present. As technology develops, studying mRNA will allow more detailed analysis of functional responses. For example, motility changes may be caused by factors unrelated to mobility that coincidentally change the abundance of organisms, and thus DNA, that utilize flagellae. This complication can be inferred from our data, as the abundance of genes involved in transcription regulation and gene expression appear to decrease in response to either one flood or both treatments. Observing the abundance of mRNA would allow us to determine if gene expression is actually decreasing. Future studies should include analyses of soil chemical properties and microbial biomass to develop a holistic understanding of the microbial ecosystems.

Several genes involved in broader functions, that is, metabolism, fatty acid metabolism, anaerobic carbon monoxide metabolism, pathogenesis, and protection, have varied results, thus broad conclusions cannot be made for these functions. Instead, our results indicate more specific responses to varying flooding frequencies that could be used as a basis for future, more targeted studies.

## 5 CONCLUSION

In this study we identify some of the impacts that increasing flooding frequency has on microbial communities and their functions. Communities appear to change significantly when they are subjected to additional floods, and functional changes reflect this. Many differences identified relate to the reduction and oxidation of substances associated with anoxia. Changes were not observed in methanogen populations, therefore as long as water drains between floods, an increase in flooding frequency is not expected to increase CH_4_ emissions (for floods lasting a couple of weeks, at least).

Conducting a laboratory experiment allows variables to be controlled and specific mechanisms tested. To more accurately represent environmental applications, further experiments in the field need to be conducted to investigate the impacts of flooding on in situ communities. Some key advantages of this would be (1) the lack of additional anthropogenic soil disturbance, (2) the inclusion of plants that act as a carbon source (among many other things), and (3) the inclusion of diurnal variations in environmental factors such as temperature and light irradiance.

## CONFLICT OF INTEREST

None declared.
