# Supplementary material for: Increasing flooding frequency alters soil microbial communities and functions under laboratory conditions
Source: Microbiologyopen. 2017 Nov 7;7(1):e00548. doi: 10.1002/mbo3.548 (PMC5822339; doi:10.1002/mbo3.548)
Supplement: Supplementary file 1 [file MBO3-7-na-s001.docx]

Supporting Information 1. Soil association.

Derived from the Academic Soils Site Report for location 392413E, 127330N, 1km x 1km (National Soil Resources Institute (NSRI), 2013).

Soil Association: Wickham 2 (711f)

*a. General Description*

Slowly permeable seasonally waterlogged fine loamy over clayey, fine silty over clayey and clayey soils. Small areas of slowly permeable calcareous soils on steeper slopes.

The major landuse on this association is defined as winter cereals and grassland in the midlands; cereals in the eastern region dairying in the South West.

*b. Distribution (England & Wales)*

The Wickham 2 association covers 1485km2 of England and Wales, which accounts for 0.98% of the landmass. The distribution of this association is shown in Supporting Information Figure 1. Note that the yellow shading represents a buffer to highlight the location of very small areas of the association.

*c. Comprising Soil Series*

Multiple soil series comprise a soil association. The soil series of the Wickham 2 association are outlined in Supporting Information Table 1 below. In some cases other minor soil series are present at a particular site, and these have been grouped together under the heading “Other”.


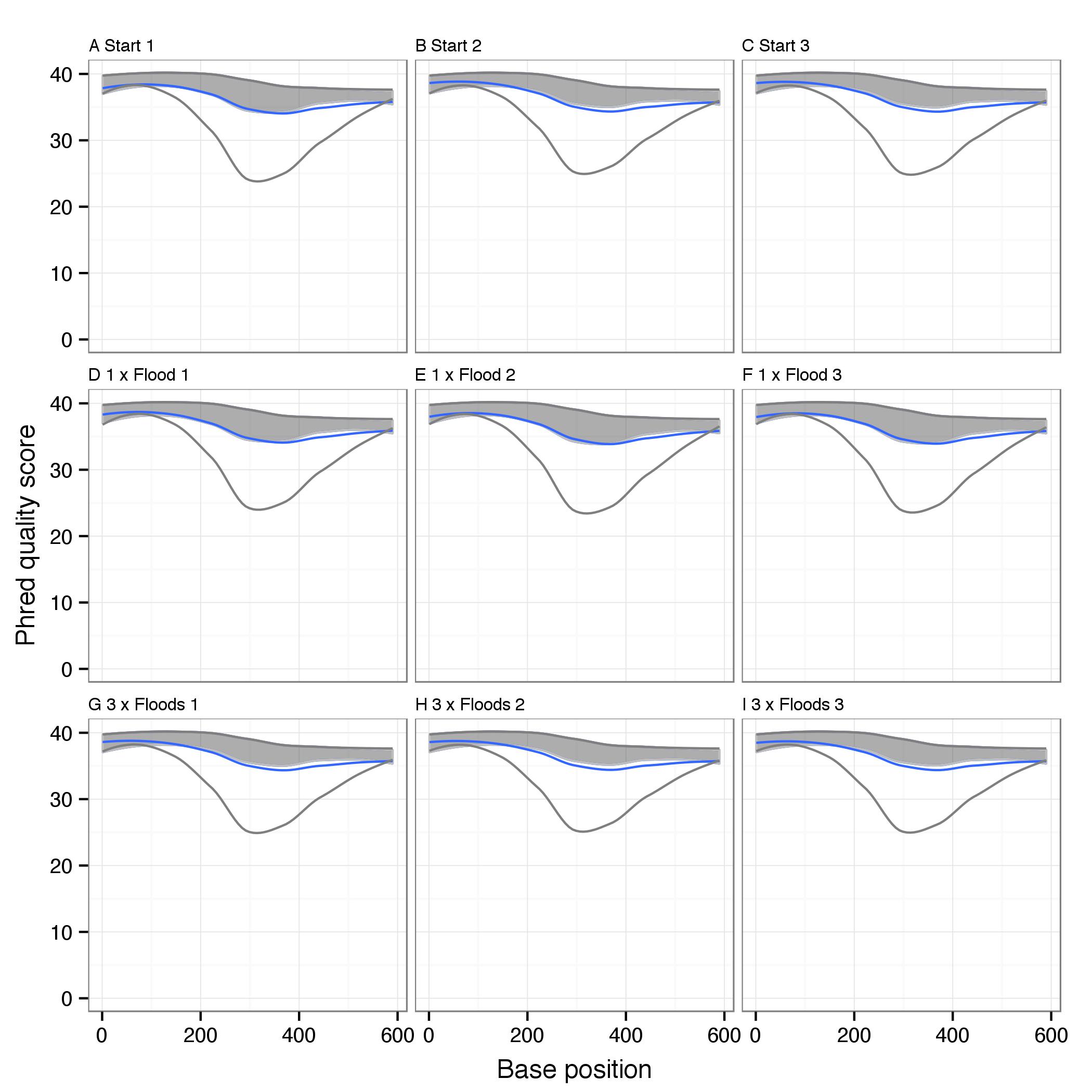


Supporting Information Figure 1. The phred quality score statistics for each base position after sequences were merged or trimmed. The dip around the 300 base-pair mark represents the end of the trimmed forward reads that were appended to the merged paired-end reads. The blue line is the mean, the shaded grey area represents the interquartile range and the grey lines represent the 10^th^ and 90^th^ percentiles.


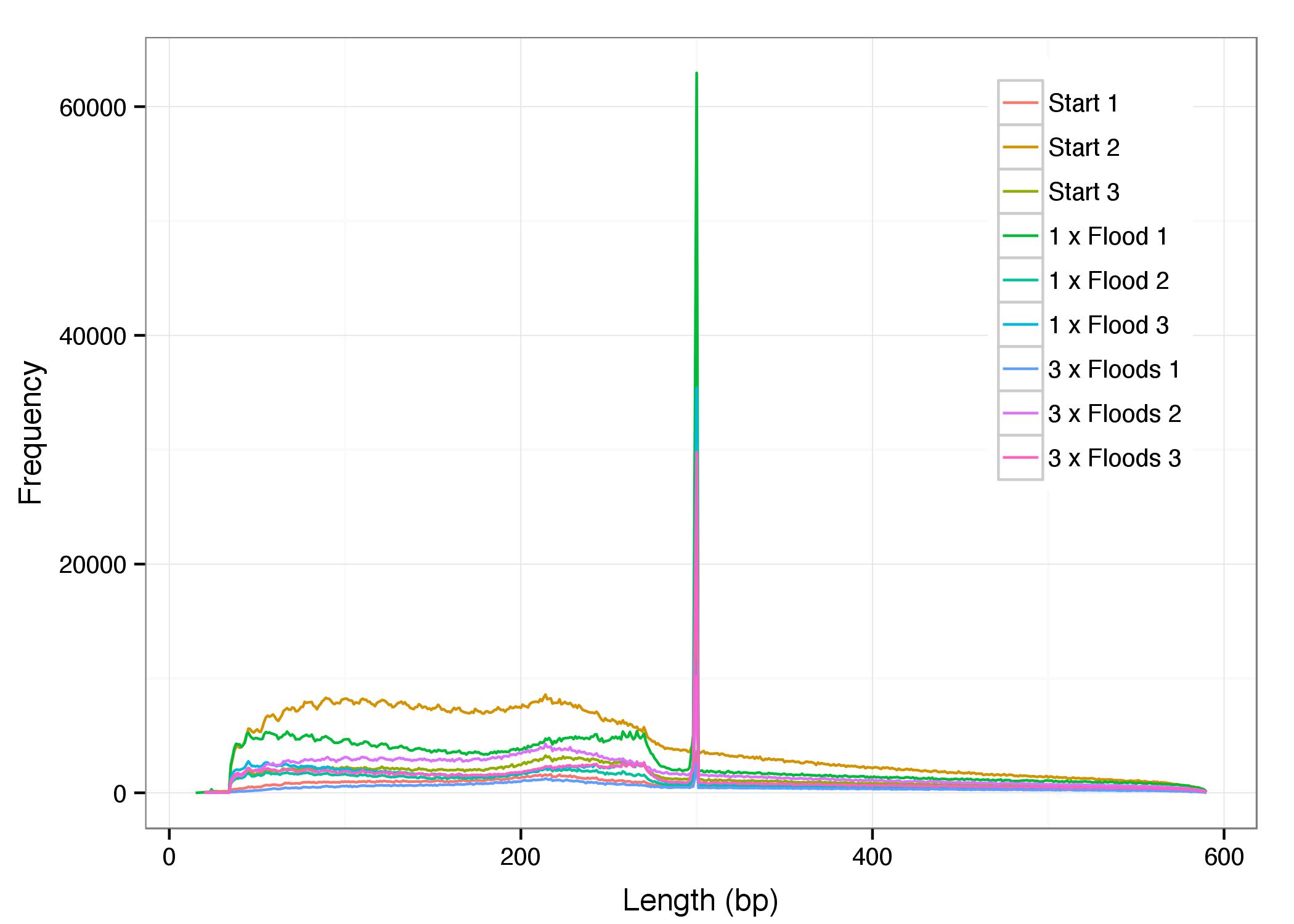


Supporting Information Figure 2. The sequence length distributions for each sample.


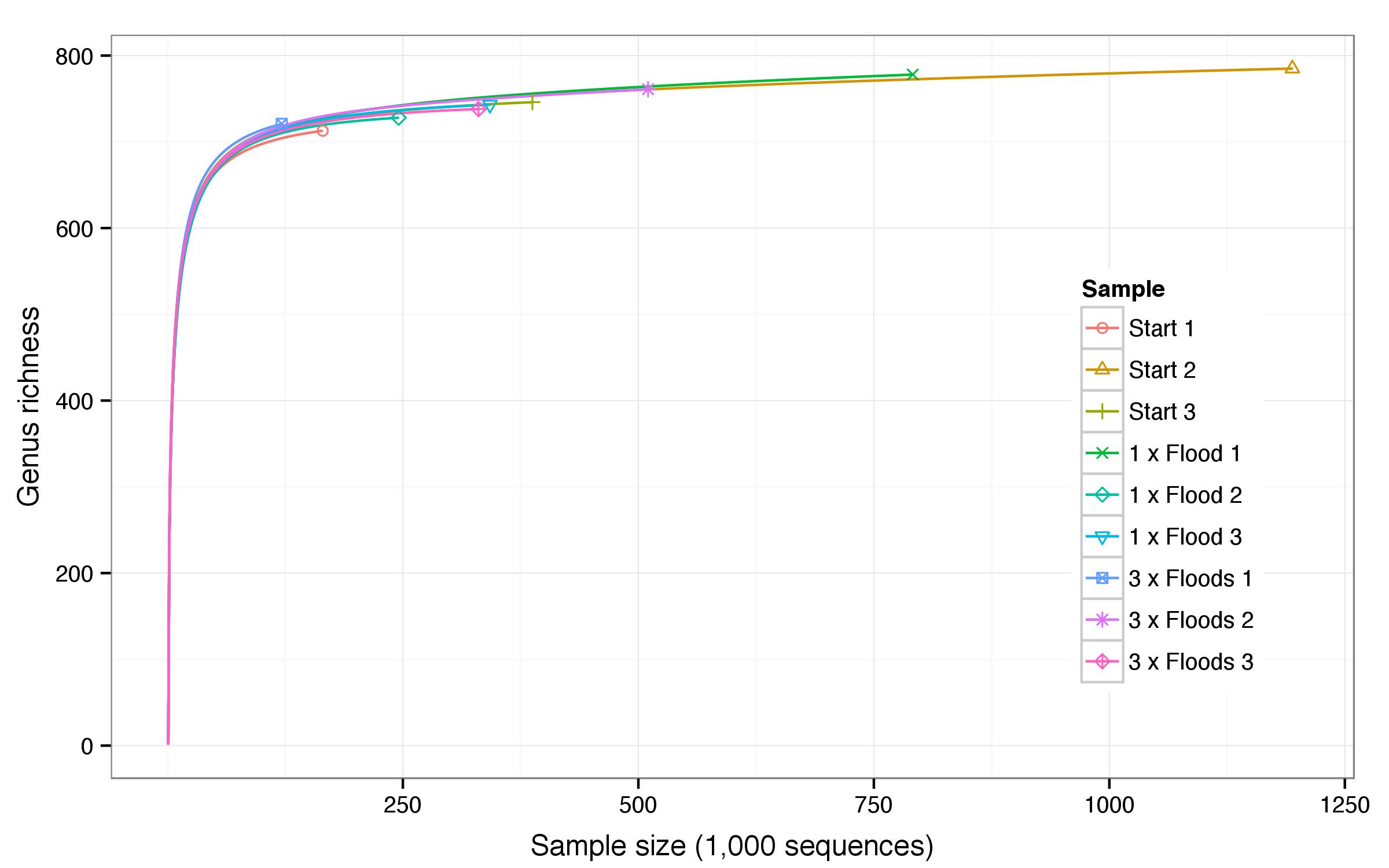


Supporting Information Figure 3. Genus rarefaction curves displaying estimates of genus richness observed per sequence. A plateauing curve signifies sufficient community coverage, where an enhanced sampling effort would not yield many additional genera.


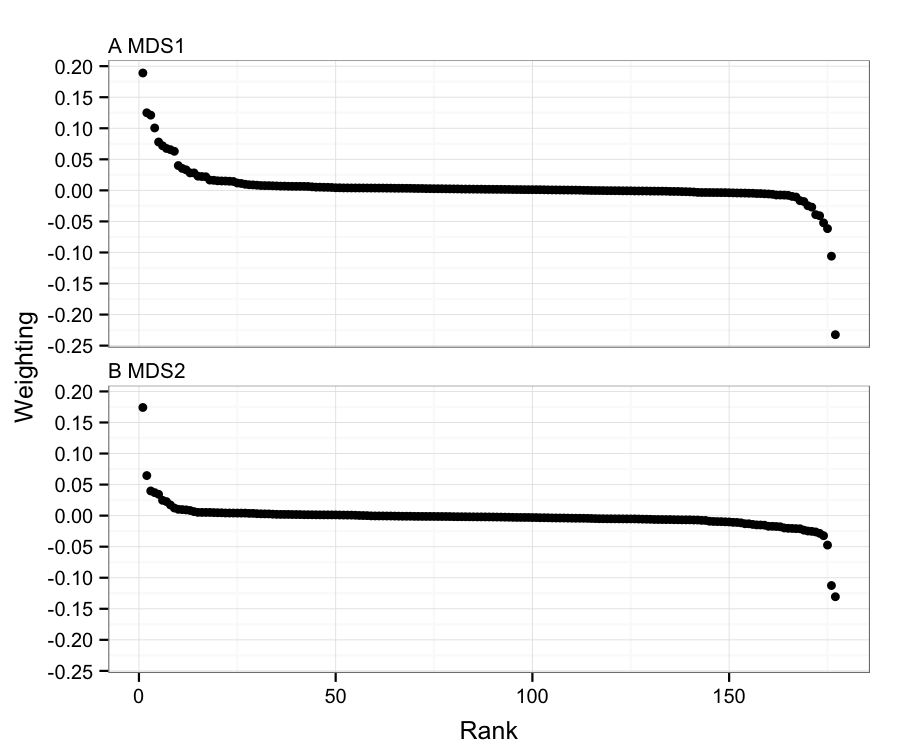


Supporting Information Figure 4. Ranked PCoA component weightings for components (MDS) 1 and 2 at the order level.

Supporting Information Table 1. Start vs. 1 Flood, Order absolute change. The greatest absolute changes in order relative abundances between the starting soil and soil that received one flood. Data for the three-flooded soil is included for comparison.

| Order | Start (x̄) | 1 x Flood (x̄) | 3 x Floods (x̄) | 1 x Flood Absolute change | 3 x Floods Absolute change |
| --- | --- | --- | --- | --- | --- |
| Actinomycetales | 2.50E-01 | 3.07E-01 | 1.98E-01 | 5.70E-02 | -5.29E-02 |
| Solibacterales | 7.00E-02 | 7.84E-02 | 1.18E-01 | 8.46E-03 | 4.79E-02 |
| Acidobacteriales | 2.73E-02 | 3.52E-02 | 4.94E-02 | 7.91E-03 | 2.21E-02 |
| Caulobacterales | 6.29E-03 | 1.12E-02 | 8.58E-03 | 4.87E-03 | 2.29E-03 |
| Myxococcales | 2.71E-02 | 3.16E-02 | 4.79E-02 | 4.51E-03 | 2.08E-02 |
| Burkholderiales | 5.20E-02 | 5.56E-02 | 6.63E-02 | 3.57E-03 | 1.43E-02 |
| Nitrosomonadales | 3.39E-03 | 5.81E-03 | 8.09E-03 | 2.42E-03 | 4.70E-03 |
| Sphingomonadales | 5.92E-03 | 8.21E-03 | 6.18E-03 | 2.29E-03 | 2.60E-04 |
| Gemmatimonadales | 3.66E-03 | 5.12E-03 | 6.63E-03 | 1.47E-03 | 2.97E-03 |
| Gallionellales | 1.14E-03 | 1.51E-03 | 5.23E-03 | 3.70E-04 | 4.09E-03 |
| Cytophagales | 5.98E-03 | 4.57E-03 | 7.19E-03 | -1.41E-03 | 1.21E-03 |
| Verrucomicrobiales | 2.56E-02 | 2.40E-02 | 3.35E-02 | -1.61E-03 | 7.95E-03 |
| Chloroflexales | 9.04E-03 | 7.41E-03 | 7.54E-03 | -1.63E-03 | -1.50E-03 |
| Rhodobacterales | 1.71E-02 | 1.52E-02 | 1.39E-02 | -1.91E-03 | -3.22E-03 |
| Chroococcales | 1.12E-02 | 9.22E-03 | 1.05E-02 | -1.93E-03 | -6.95E-04 |
| Ktedonobacterales | 1.02E-02 | 7.93E-03 | 6.55E-03 | -2.23E-03 | -3.60E-03 |
| Sphingobacteriales | 6.29E-03 | 3.91E-03 | 6.53E-03 | -2.38E-03 | 2.34E-04 |
| Rhodospirillales | 2.09E-02 | 1.69E-02 | 1.50E-02 | -4.01E-03 | -5.88E-03 |
| Planctomycetales | 3.99E-02 | 2.11E-02 | 2.41E-02 | -1.88E-02 | -1.58E-02 |
| Rhizobiales | 2.07E-01 | 1.69E-01 | 1.54E-01 | -3.85E-02 | -5.28E-02 |

Supporting Information Table 2. Start vs. 3 Floods, Order absolute change. The greatest absolute changes in order relative abundances between the starting soil and soil that received three floods. Data for the single-flood soil is included for comparison.

| Order | Start (x̄) | 1 x Flood (x̄) | 3 x Floods (x̄) | 1 x Flood Absolute change | 3 x Floods Absolute change |
| --- | --- | --- | --- | --- | --- |
| Solibacterales | 7.00E-02 | 7.84E-02 | 1.18E-01 | 8.46E-03 | 4.79E-02 |
| Acidobacteriales | 2.73E-02 | 3.52E-02 | 4.94E-02 | 7.91E-03 | 2.21E-02 |
| Myxococcales | 2.71E-02 | 3.16E-02 | 4.79E-02 | 4.51E-03 | 2.08E-02 |
| Burkholderiales | 5.20E-02 | 5.56E-02 | 6.63E-02 | 3.57E-03 | 1.43E-02 |
| Verrucomicrobiales | 2.56E-02 | 2.40E-02 | 3.35E-02 | -1.61E-03 | 7.95E-03 |
| Desulfuromonadales | 1.25E-02 | 1.24E-02 | 1.92E-02 | -1.27E-04 | 6.68E-03 |
| Nitrosomonadales | 3.39E-03 | 5.81E-03 | 8.09E-03 | 2.42E-03 | 4.70E-03 |
| Gallionellales | 1.14E-03 | 1.51E-03 | 5.23E-03 | 3.70E-04 | 4.09E-03 |
| Gemmatimonadales | 3.66E-03 | 5.12E-03 | 6.63E-03 | 1.47E-03 | 2.97E-03 |
| Bacteroidales | 3.21E-03 | 2.74E-03 | 6.14E-03 | -4.71E-04 | 2.93E-03 |
| Bacillales | 1.53E-02 | 1.40E-02 | 1.41E-02 | -1.27E-03 | -1.17E-03 |
| Rubrobacterales | 4.79E-03 | 4.44E-03 | 3.38E-03 | -3.44E-04 | -1.40E-03 |
| Chloroflexales | 9.04E-03 | 7.41E-03 | 7.54E-03 | -1.63E-03 | -1.50E-03 |
| Rhodobacterales | 1.71E-02 | 1.52E-02 | 1.39E-02 | -1.91E-03 | -3.22E-03 |
| Ktedonobacterales | 1.02E-02 | 7.93E-03 | 6.55E-03 | -2.23E-03 | -3.60E-03 |
| Solirubrobacterales | 1.66E-02 | 1.67E-02 | 1.16E-02 | 5.64E-05 | -5.03E-03 |
| Rhodospirillales | 2.09E-02 | 1.69E-02 | 1.50E-02 | -4.01E-03 | -5.88E-03 |
| Planctomycetales | 3.99E-02 | 2.11E-02 | 2.41E-02 | -1.88E-02 | -1.58E-02 |
| Rhizobiales | 2.07E-01 | 1.69E-01 | 1.54E-01 | -3.85E-02 | -5.28E-02 |
| Actinomycetales | 2.50E-01 | 3.07E-01 | 1.98E-01 | 5.70E-02 | -5.29E-02 |

Supporting Information Table 3. Start vs. 1 Flood, Order fold change. The greatest fold changes in order relative abundances between the starting soil and soil that received one flood. Data for the three-flooded soil is included for comparison.

| Order | Start (x̄) | 1 x Flood (x̄) | 3 x Floods (x̄) | 1 x Flood Fold change | 3 x Floods Fold change |
| --- | --- | --- | --- | --- | --- |
| Marchantiales | 3.00E-07 | 3.46E-06 | 9.90E-06 | 11.535 | 33.056 |
| Bangiales | 3.00E-07 | 2.82E-06 | 0.00E+00 | 9.416 | 0.000 |
| Euglyphida | 2.40E-06 | 9.71E-06 | 1.54E-05 | 4.052 | 6.424 |
| Entomoplasmatales | 8.99E-07 | 2.82E-06 | 7.24E-06 | 3.139 | 8.049 |
| Spirurida | 5.19E-05 | 1.14E-04 | 7.24E-05 | 2.200 | 1.395 |
| Hymenostomatida | 2.22E-05 | 4.57E-05 | 5.26E-05 | 2.061 | 2.372 |
| Haemosporida | 3.78E-05 | 6.88E-05 | 3.98E-05 | 1.820 | 1.053 |
| Caulobacterales | 6.29E-03 | 1.12E-02 | 8.58E-03 | 1.774 | 1.364 |
| Nitrosomonadales | 3.39E-03 | 5.81E-03 | 8.09E-03 | 1.713 | 2.383 |
| Peniculida | 3.65E-05 | 6.21E-05 | 7.53E-05 | 1.703 | 2.066 |
| Coleochaetales | 5.99E-07 | 0.00E+00 | 3.06E-06 | 0.000 | 5.109 |
| Asparagales | 9.20E-07 | 0.00E+00 | 0.00E+00 | 0.000 | 0.000 |
| Caryophyllales | 9.20E-07 | 0.00E+00 | 7.19E-07 | 0.000 | 0.782 |
| Cyrtocrinida | 9.20E-07 | 0.00E+00 | 0.00E+00 | 0.000 | 0.000 |
| Synurales | 9.20E-07 | 0.00E+00 | 0.00E+00 | 0.000 | 0.000 |
| Eupodiscales | 2.20E-06 | 0.00E+00 | 7.19E-07 | 0.000 | 0.327 |
| Polypodiales | 2.20E-06 | 0.00E+00 | 0.00E+00 | 0.000 | 0.000 |
| Pseudoscourfieldiales | 2.44E-06 | 0.00E+00 | 1.11E-06 | 0.000 | 0.456 |
| Fabales | 3.10E-06 | 0.00E+00 | 0.00E+00 | 0.000 | 0.000 |

Supporting Information Table 4. Start vs. 3 Floods, Order fold change. The greatest fold changes in order relative abundances between the starting soil and soil that received three floods. Data for the single-flood soil is included for comparison.

| Order | Start (x̄) | 1 x Flood (x̄) | 3 x Floods (x̄) | 1 x Flood Fold change | 3 x Floods Fold change |
| --- | --- | --- | --- | --- | --- |
| Marchantiales | 3.00E-07 | 3.46E-06 | 9.90E-06 | 11.535 | 33.056 |
| Zygnematales | 3.00E-07 | 0.00E+00 | 3.06E-06 | 0.000 | 10.218 |
| Entomoplasmatales | 8.99E-07 | 2.82E-06 | 7.24E-06 | 3.139 | 8.049 |
| Euglyphida | 2.40E-06 | 9.71E-06 | 1.54E-05 | 4.052 | 6.424 |
| Coleochaetales | 5.99E-07 | 0.00E+00 | 3.06E-06 | 0.000 | 5.109 |
| Gallionellales | 1.14E-03 | 1.51E-03 | 5.23E-03 | 1.326 | 4.602 |
| Coleoptera | 1.70E-05 | 2.52E-05 | 5.12E-05 | 1.488 | 3.019 |
| Pyrenomonadales | 3.00E-06 | 3.56E-06 | 7.84E-06 | 1.189 | 2.615 |
| Fibrobacterales | 1.03E-04 | 1.29E-04 | 2.66E-04 | 1.252 | 2.576 |
| Chlorellales | 1.22E-06 | 4.53E-07 | 3.06E-06 | 0.371 | 2.510 |
| Chlorokybales | 9.20E-07 | 1.50E-06 | 0.00E+00 | 1.632 | 0.000 |
| Echinorhynchida | 9.20E-07 | 4.53E-07 | 0.00E+00 | 0.492 | 0.000 |
| Asparagales | 9.20E-07 | 0.00E+00 | 0.00E+00 | 0.000 | 0.000 |
| Cyrtocrinida | 9.20E-07 | 0.00E+00 | 0.00E+00 | 0.000 | 0.000 |
| Synurales | 9.20E-07 | 0.00E+00 | 0.00E+00 | 0.000 | 0.000 |
| Glomerales | 1.52E-06 | 1.05E-06 | 0.00E+00 | 0.691 | 0.000 |
| Polypodiales | 2.20E-06 | 0.00E+00 | 0.00E+00 | 0.000 | 0.000 |
| Vaucheriales | 2.50E-06 | 9.05E-07 | 0.00E+00 | 0.362 | 0.000 |
| Fabales | 3.10E-06 | 0.00E+00 | 0.00E+00 | 0.000 | 0.000 |
| Capnodiales | 6.54E-06 | 1.46E-06 | 0.00E+00 | 0.224 | 0.000 |

Supporting Information Table 5. Start vs. 1 Flood, Level 3 function absolute change. The greatest absolute changes in level 2 function relative abundances between the starting soil and soil that received one flood. Data for the three-flooded soil is included for comparison.

| Function | Start (x̄) | 1 x Flood (x̄) | 3 x Floods (x̄) | 1 x Flood Absolute change | 3 x Floods Absolute change |
| --- | --- | --- | --- | --- | --- |
| Ton and Tol transport systems | 3.20E-03 | 3.72E-03 | 4.59E-03 | 5.25E-04 | 1.39E-03 |
| Fatty acid degradation regulons | 7.08E-03 | 7.55E-03 | 6.68E-03 | 4.74E-04 | -3.93E-04 |
| Iron acquisition in Vibrio | 1.96E-03 | 2.38E-03 | 2.81E-03 | 4.19E-04 | 8.48E-04 |
| Biotin biosynthesis | 4.96E-03 | 5.37E-03 | 4.75E-03 | 4.04E-04 | -2.10E-04 |
| CBSS-316057.3.peg.1308 | 2.61E-03 | 3.01E-03 | 2.56E-03 | 4.02E-04 | -5.35E-05 |
| Fatty acid metabolism cluster | 6.04E-03 | 6.40E-03 | 5.66E-03 | 3.62E-04 | -3.74E-04 |
| n-Phenylalkanoic acid degradation | 6.04E-03 | 6.40E-03 | 5.66E-03 | 3.61E-04 | -3.76E-04 |
| Sugar utilization in Thermotogales | 1.29E-02 | 1.32E-02 | 1.45E-02 | 3.48E-04 | 1.63E-03 |
| Lysine fermentation | 3.60E-03 | 3.94E-03 | 3.63E-03 | 3.41E-04 | 2.96E-05 |
| Leucine Degradation and HMG-CoA Metabolism | 3.39E-03 | 3.69E-03 | 3.38E-03 | 2.99E-04 | -9.14E-06 |
| Universal GTPases | 5.20E-03 | 4.90E-03 | 5.22E-03 | -2.98E-04 | 1.98E-05 |
| Sulfatases and sulfatase modifying factor 1 | 3.72E-03 | 3.42E-03 | 3.40E-03 | -3.00E-04 | -3.20E-04 |
| RNA polymerase bacterial | 3.06E-03 | 2.76E-03 | 2.95E-03 | -3.02E-04 | -1.10E-04 |
| Respiratory Complex I | 4.87E-03 | 4.54E-03 | 5.34E-03 | -3.29E-04 | 4.67E-04 |
| Alkanesulfonate assimilation | 3.80E-03 | 3.38E-03 | 3.28E-03 | -4.28E-04 | -5.22E-04 |
| Phage integration and excision | 5.23E-03 | 4.79E-03 | 4.17E-03 | -4.34E-04 | -1.05E-03 |
| ABC transporter branched-chain amino acid (TC 3.A.1.4.1) | 3.73E-03 | 3.18E-03 | 3.14E-03 | -5.48E-04 | -5.90E-04 |
| cAMP signaling in bacteria | 8.19E-03 | 6.78E-03 | 6.22E-03 | -1.42E-03 | -1.97E-03 |
| CBSS-222523.1.peg.1311 | 7.83E-03 | 6.36E-03 | 5.91E-03 | -1.46E-03 | -1.91E-03 |
| Iojap | 1.00E-02 | 8.29E-03 | 8.14E-03 | -1.71E-03 | -1.86E-03 |

Supporting Information Table 6. Start vs. 3 Floods, Level 3 function absolute change. The greatest absolute changes in level 2 function relative abundances between the starting soil and soil that received three floods. Data for the single-flood soil is included for comparison.

| Function | Start (x̄) | 1 x Flood (x̄) | 3 x Floods (x̄) | 1 x Flood Absolute change | 3 x Floods Absolute change |
| --- | --- | --- | --- | --- | --- |
| Sugar utilization in Thermotogales | 1.29E-02 | 1.32E-02 | 1.45E-02 | 3.48E-04 | 1.63E-03 |
| Ton and Tol transport systems | 3.20E-03 | 3.72E-03 | 4.59E-03 | 5.25E-04 | 1.39E-03 |
| Cobalt-zinc-cadmium resistance | 5.34E-03 | 5.05E-03 | 6.67E-03 | -2.95E-04 | 1.33E-03 |
| Bacterial Chemotaxis | 2.26E-03 | 2.32E-03 | 3.15E-03 | 6.08E-05 | 8.89E-04 |
| Flagellar motility | 1.74E-03 | 2.00E-03 | 2.61E-03 | 2.61E-04 | 8.64E-04 |
| Iron acquisition in Vibrio | 1.96E-03 | 2.38E-03 | 2.81E-03 | 4.19E-04 | 8.48E-04 |
| Lactose and Galactose Uptake and Utilization | 2.67E-03 | 2.69E-03 | 3.33E-03 | 1.75E-05 | 6.60E-04 |
| Hydrogenases | 1.34E-03 | 1.45E-03 | 1.99E-03 | 1.09E-04 | 6.56E-04 |
| Flagellum | 3.05E-03 | 3.29E-03 | 3.66E-03 | 2.41E-04 | 6.10E-04 |
| C jejuni colonization of chick caeca | 1.79E-03 | 1.92E-03 | 2.31E-03 | 1.30E-04 | 5.18E-04 |
| Serine-glyoxylate cycle | 1.53E-02 | 1.51E-02 | 1.48E-02 | -2.01E-04 | -5.05E-04 |
| Alkanesulfonate assimilation | 3.80E-03 | 3.38E-03 | 3.28E-03 | -4.28E-04 | -5.22E-04 |
| ABC transporter branched-chain amino acid (TC 3.A.1.4.1) | 3.73E-03 | 3.18E-03 | 3.14E-03 | -5.48E-04 | -5.90E-04 |
| Trehalose Biosynthesis | 5.52E-03 | 5.48E-03 | 4.85E-03 | -4.17E-05 | -6.78E-04 |
| Phage integration and excision | 5.23E-03 | 4.79E-03 | 4.17E-03 | -4.34E-04 | -1.05E-03 |
| CBSS-314269.3.peg.1840 | 3.07E-03 | 2.86E-03 | 1.98E-03 | -2.07E-04 | -1.09E-03 |
| CO Dehydrogenase | 3.19E-03 | 3.02E-03 | 2.10E-03 | -1.68E-04 | -1.09E-03 |
| Iojap | 1.00E-02 | 8.29E-03 | 8.14E-03 | -1.71E-03 | -1.86E-03 |
| CBSS-222523.1.peg.1311 | 7.83E-03 | 6.36E-03 | 5.91E-03 | -1.46E-03 | -1.91E-03 |
| cAMP signaling in bacteria | 8.19E-03 | 6.78E-03 | 6.22E-03 | -1.42E-03 | -1.97E-03 |
